# Monovalent Copper Cation Doping Enables High-Performance CsPbIBr_2_-Based All-Inorganic Perovskite Solar Cells

**DOI:** 10.3390/nano12234317

**Published:** 2022-12-05

**Authors:** Zhaonan Du, Huimin Xiang, Amin Xie, Ran Ran, Wei Zhou, Wei Wang, Zongping Shao

**Affiliations:** 1 State Key Laboratory of Materials-Oriented Chemical Engineering, College of Chemical Engineering, Nanjing Tech University, Nanjing 210009, China; 2 WA School of Mines: Minerals, Energy and Chemical Engineering, Curtin University, Perth, WA 6845, Australia

**Keywords:** CsPbIBr_2_, perovskite solar cells, all-inorganic perovskites, Cu^+^ doping, stability

## Abstract

Organic–inorganic perovskite solar cells (PSCs) have delivered the highest power conversion efficiency (PCE) of 25.7% currently, but they are unfortunately limited by several key issues, such as inferior humid and thermal stability, significantly retarding their widespread application. To tackle the instability issue, all-inorganic PSCs have attracted increasing interest due to superior structural, humid and high-temperature stability to their organic–inorganic counterparts. Nevertheless, all-inorganic PSCs with typical CsPbIBr_2_ perovskite as light absorbers suffer from much inferior PCEs to those of organic–inorganic PSCs. Functional doping is regarded as a simple and useful strategy to improve the PCEs of CsPbIBr_2_-based all-inorganic PSCs. Herein, we report a monovalent copper cation (Cu^+^)-doping strategy to boost the performance of CsPbIBr_2_-based PSCs by increasing the grain sizes and improving the CsPbIBr_2_ film quality, reducing the defect density, inhibiting the carrier recombination and constructing proper energy level alignment. Consequently, the device with optimized Cu^+^-doping concentration generates a much better PCE of 9.11% than the pristine cell (7.24%). Moreover, the Cu^+^ doping also remarkably enhances the humid and thermal durability of CsPbIBr_2_-based PSCs with suppressed hysteresis. The current study provides a simple and useful strategy to enhance the PCE and the durability of CsPbIBr_2_-based PSCs, which can promote the practical application of perovskite photovoltaics.

## 1. Introduction

The energy crisis and greenhouse gas emissions are the most crucial worldwide problems nowadays, which are mainly caused by the low efficiency and excessive consumption of nonrenewable fossil fuels [1,2]. Thus, the utilization of renewable energies and the development of relevant energy conversion technologies are highly urgent and essential [3,4,5,6,7]. Among various types of renewable energies/sources, solar energy has gained particular interest due to its inexhaustible and clean nature, which can be efficiently utilized by three main routes: photovoltaic cells, photocatalysis and solar-thermal power generation [1,8,9,10]. In particular, photovoltaic cells (solar cells) have received increasing attention and achieved significant progress in the past decades because of their direct and efficient transformation of sunlight energy into electricity [11,12,13,14,15]. Nowadays, the commercial photovoltaic markets are dominated by silicon-based solar cells, showing excellent stability and high PCE of 27.6% [16]. However, silicon-based solar cells are limited by high-cost and complex fabrication procedures, and toxic by-products in the silicon manufacturing, which are harmful to the environment [17,18]. Consequently, the development of new-type solar cells with simple, cost-effective and environmentally friendly fabrication processes is highly crucial [19,20].

Among various third-generation solar cells, perovskite solar cells (PSCs) with halide perovskites as light absorbers are regarded as more attractive than dye-sensitized and organic solar cells due to the cheap raw materials used, the simple and low-cost fabrication procedures, as well as their rapidly increasing power conversion efficiencies (PCEs) in the last 10 years [21,22]. The theoretical limit of the PCE has been known for PSCs and is approximately 30% [23]. Since the invention of PSCs by Miyasaka et al. in 2009, typical organic–inorganic PSCs have witnessed a rapid increase in their PCEs from only 3.8% to over 25% due to several unique advantages of organic–inorganic halide perovskites, such as proper and adjustable band gaps, large light absorption coefficient, long diffusion length and superior mobility of carriers [24,25,26,27]. The general formula of halide perovskites is ABX_3_, where A is monovalent cations including CH_3_NH_3_^+^ (MA^+^), CH_3_(NH_2_)_2_^+^ (FA^+^) and cesium (Cs^+^), B is divalent cations including Pb^2+^, Sn^2+^ and Ge^2+^, while X represents the halogen anions including I^−^, Br^−^ and Cl^−^ [28,29,30]. It is well accepted that in addition to the PCEs, the long-term durability under humid, high-temperature and sunlight irradiation conditions is another key prerequisite for the widespread application of PSCs [31,32]. The plasmonic effect plays a very effective role in improving the efficiency of perovskite cells. By optimizing the adaptation of the plasmon photovoltaic channel, the relative efficiency was enhanced 40% [33]. Nevertheless, organic–inorganic hybrid PSCs suffer from inferior stability due to the high sensitivity of organic–inorganic perovskites against humidity, heat and light illumination, and they can be decomposed into PbI_2_ because of the inherent volatility and hygroscopicity of organic A-site cations (e.g., MA^+^, FA^+^) in the organic–inorganic perovskites [34,35,36,37].

All-inorganic halide perovskites are considered as attractive alternatives to their organic–inorganic counterparts to tackle the above-mentioned instability issue due to having superior intrinsic structural stability and much lower sensitivity against humidity and heat [38,39]. During the past seven years, CsPbI_x_Br_3−x_ (0 ≤ x ≤ 3) as an important class of all-inorganic halide perovskites, has received particular attention as an efficient light absorber for PSCs due to the tunable band gaps, high intrinsic stability and superior carrier transport capability, etc. [40,41]. For instance, the PCEs of CsPbI_3_-based PSCs with a proper band gap of 1.73 eV have reached 21% nowadays. However, CsPbI_3_ perovskites suffer from the detrimental phase transformation from black CsPbI_3_ non-perovskite yellow phase with a much wider band gap (3.01 eV) at room temperature, exhibiting negative impacts on the PCEs and the stability of CsPbI_3_-based PSCs [42,43]. In contrast, CsPbBr_3_ perovskites have exhibited excellent phase structural stability and high heat/moisture tolerance, which suffered from the extremely large band gap (2.3 eV), remarkably limiting sunlight absorption range and then the cell performance [44,45]. On this basis, mixed halide all-inorganic CsPbIBr_2_ perovskite has attracted extensive interest due to the well-balanced band gap value (2.05 eV) and phase structural stability [46,47].

For single-junction PSCs, the calculated Shockley–Queisser limit is approximately 33%. Various approaches such as greatly increasing grain size, significantly reducing defect amount and band gaps, significantly improving carrier transport and separation capability, as well as constructing tandem PSCs, may allow approaching or surpassing the Shockley–Queisser limits of PSCs [3,48,49,50]. Nowadays, the PCEs of CsPbIBr_2_-based PSCs have exceeded 12%, which is much inferior to the organic–inorganic PSCs [51,52]. It has been reported that the limited PCEs of CsPbIBr_2_-based cells are mainly caused by the inferior quality CsPbIBr_2_ films fabricated by the conventional solution route, exhibiting numerous pinholes, grain boundaries and defects, which functioned as recombination sites for photoinduced carriers to suppress the cell performance [53,54]. Therefore, a variety of strategies have been devoted to achieving high-quality CsPbIBr_2_ light-absorbing films, such as interfacial modification, functional/selective cation doping, post-treatment process and additive engineering, etc. [55,56,57]. Among these available strategies, cation doping/substitution is widely employed to tailor the optical/electronic properties and film quality of CsPbIBr_2_, which plays significant roles in creating homogeneous nucleation sites to control the crystallization and growth of CsPbIBr_2_ crystals, contributing to remarkably enhanced perovskite film quality [58,59,60]. Nowadays, the cation doping in CsPbIBr_2_ is mainly focused on the utilization of divalent metal cations to substitute Pb^2+^, including Sn^2+^, Cu^2+^, Zn^2+^, Ba^2+^, Eu^2+^, etc., which effectively enhance the crystallinity and morphology of CsPbIBr_2_ films [61,62,63,64,65,66]. For instance, Sn^2+^ plays a crucial role in regulating the band gap and enhancing the film quality of CsPbIBr_2_, and Zhao et al. have reported that remarkably enhanced PCE (11.33%) and long-term stability were achieved by Sn^2+^ doping in CsPbIBr_2_-based PSCs [61,64]. In our previous work, we have found that Cu^2+^ doping in CsPbIBr_2_ with optimized doping concentration effectively inhibited the carrier recombination by reducing the defect amount, and it enhanced the perovskite film quality by improving the crystallinity and enlarging the grain sizes, thereby remarkably improving the PCEs and humid/thermal stability of CsPbIBr_2_-based PSCs [62].

Nevertheless, it should be noted that the monovalent cation doping in the B-site of CsPbIBr_2_ is much less investigated, which needs further exploration. Herein, we have employed monovalent copper cations (Cu^+^) as effective B-site dopants for CsPbIBr_2_ perovskites, which effectively improved the CsPbIBr_2_ film quality in terms of a suppressed amount of grain boundaries, increased crystallinity and grain sizes, passivated surface defects and inhibited interfacial carrier recombination. After optimizing the Cu^+^ doping concentration, the CsPbIBr_2_-0.50%Cu cell produced a superb PCE of 9.11%, 25.8% higher than that of the pristine cell. Furthermore, Cu^+^ doping also remarkably improved the high-temperature and humid durability of CsPbIBr_2_-based PSCs with reduced hysteresis effect. Our current work can present some important insights for the design of high-performance all-inorganic PSCs, which may promote the widespread application of perovskite photovoltaics.

## 2. Materials and Methods

F^−^-doped tin oxide (FTO) glasses (2.2-mm thick and 7 Ω sq^−1^) were first cleaned by various solvents, dried by N_2_ and further treated by oxygen plasma cleaning [56]. Detailed information about the fabrication of CsPbIBr_2_-based PSCs with a configuration of compact TiO_2_ (c-TiO_2_)/perovskite/2,2′,7,7′-tetrakis (N,N-di-p-methoxyphenylamine)-9,9′-spirobifluorene (Spiro-OMeTAD)/Ag can be found in our previous work [62]. As for the Cu^+^-doped CsPbIBr_2_-based cells, different amounts of as-prepared 0.2 M cuprous bromide/dimethyl sulfoxide (CuBr/DMSO) solution were added into the 1 M CsPbIBr_2_ precursor solution to prepare various Cu^+^-doped CsPbIBr_2_ films at fixed molar ratios of 0.25, 0.50 and 0.75%. The effective area of the cell was 0.0625 cm^2^ in this work. For the thermal stability test, the carbon electrode was deposited by doctor blade for the hole-transporting layer (HTL)-free CsPbIBr_2_-based PSCs and the detailed information can be found in our previous work [62].

X-ray diffraction (XRD) patterns of various samples were acquired by X-ray diffractometer (Rigaku Smartlab, Matsubara-cho, Tokyo, Japan) with Cu*Kα* radiation. X-ray/ultraviolet photoelectron spectroscopy (XPS/UPS) profiles of various samples were acquired by XPS spectrometer (PHI5600 Versa Probe, Brooklyn, NY, USA). Energy-dispersive X-ray spectroscopy (EDX, Octane Ultra, Lafayette, LA, USA) and scanning electron microscope (SEM, Hitachi S-4800, Chiyoda-ku, Tokyo, Japan) were employed to investigate the microstructures and elemental mapping of various films. The surface roughness values of various perovskite films were acquired by atomic force microscopy (AFM, SmartSPM, Horiba, Minami-ku, Tokyo, Japan). Ultraviolet–visible (UV-vis) and photoluminescence (PL) spectra were obtained by Lambda 750 s spectrometer (PerkinElmer, Waltham, MA, USA) and fluorescence spectrometer (PerkinElmer, FL 6500, Waltham, MA, USA), respectively. Photocurrent density-voltage (*J−V*) curves of various cells were acquired by Zolix solar simulator under 1 Sun irradiation (100 mW cm^−2^) after calibration. Electrochemical impedance spectroscopy (EIS) and external quantum efficiency (EQE) and spectra of various cells were acquired by electrochemical workstation (CHI760E) and Zolix Solar Cell Scan 100 instrument, respectively.

## 3. Results and Discussion

**Figure 1:**
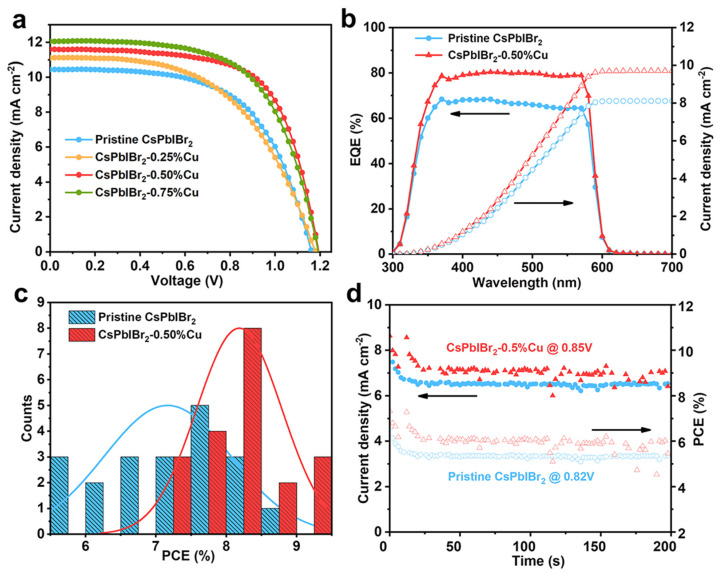
(**a**) *J−V* curves of CsPbIBr_2_ and various Cu^+^-doped CsPbIBr_2_ cells under a reverse scan direction. (**b**) EQE profiles, integrated photocurrent densities based on CsPbIBr_2_ and CsPbIBr_2_-0.50%Cu devices, (**c**) statistical PCE distributions of PSCs based on CsPbIBr_2_ and CsPbIBr_2_-0.50%Cu, (**d**) steady PCE and J_sc_ values of CsPbIBr_2_ and CsPbIBr_2_-0.50%Cu cells.

Cu^+^ cations with three different molar ratios of 0.25, 0.50 and 0.75% were doped into CsPbIBr_2_, which were labeled as CsPbIBr_2_, CsPbIBr_2_-0.25%Cu, CsPbIBr_2_-0.50%Cu and CsPbIBr_2_-0.75%Cu, respectively. Figure S1 displays the typical cross-sectional SEM image of as-fabricated CsPbIBr_2_-0.50%Cu cell and it was found that the 350 nm-thick perovskite film was firmly adhered on the surface of c-TiO_2_ film. Based on the *J−V* curves of various CsPbIBr_2_ cells, as depicted in Figure 1a and relevant photovoltaic parameters in Figure S2, the Cu^+^ doping in CsPbIBr_2_ significantly boosted the PCEs of corresponding cells by increasing the fill factor (FF) and short-circuit current density (*J*_sc_). More specifically, the unmodified cell displayed a champion PCE of 7.24% with an open-circuit voltage (*V*_oc_) of 1.16 V, a *J*_sc_ of 10.4 mA cm^−2^ and an FF of 0.597. In addition, the cell performance of Cu^+^-doped CsPbIBr_2_-based PSCs exhibited a volcano-like trend with increased Cu^+^ doping contents, suggesting that the introduction of a suitable concentration of Cu^+^ cations significantly enhanced the PCEs of CsPbIBr_2_ cells. Particularly, the CsPbIBr_2_-0.50%Cu cell displayed the highest PCE of 9.11%, with a *V*_oc_ of 1.19 V, a *J*_sc_ of 11.6 mA cm^−2^ and a FF of 0.658 (Table S1). The CsPbIBr_2_-0.75%Cu cell exhibited a reduced PCE, which was mainly attributed to decreased FF value induced by the poor quality of CsPbIBr_2_ film and higher defect concentration, which will be discussed later. Based on the EQE spectra in Figure 1b, the CsPbIBr_2_-0.50%Cu cell displayed higher EQE values than those of the pristine cell at a wavelength range of 300 to 600 nm. In addition, CsPbIBr_2_-0.50%Cu cell generated a larger integrated *J*_sc_ value of 9.7 mA cm^−2^ than that of the pristine cell (8.1 mA cm^−2^), which was consistent with the *J−V* results (Figure 1a). To confirm the universality of such PCE enhancement, 20 devices for each type of PSC (without and with 0.50%Cu^+^ substitution) were fabricated and tested with the photovoltaic parameter distributions displayed in Figure 1c. It is clear that the average PCE of the CsPbIBr_2_-0.50%Cu cell was much larger than that of the unmodified device due to the significantly improved *J*_sc_ and FF values (Figure S2). In addition, based on the maximum power point tracking (MPPT) profiles of PSCs as depicted in Figure 1d, the CsPbIBr_2_-0.50%Cu cell produced much larger stabilized PCE and *J*_sc_ values than those of the pristine CsPbIBr_2_ cell at the maximum power point.

**Figure 2:**
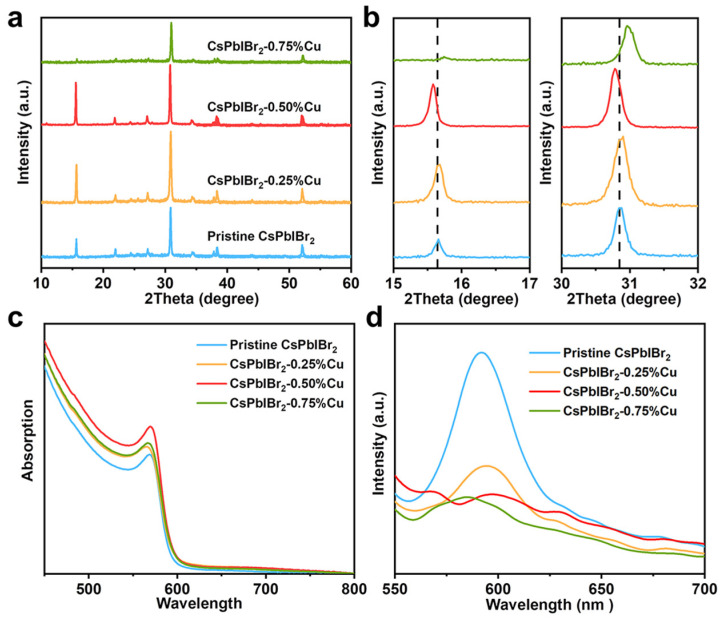
(**a**) XRD patterns, (**b**) magnified (100) and (200) peaks, (**c**) UV-vis and (**d**) PL spectra (deposited on conductive substrate) of CsPbIBr_2_ and different Cu^+^-doped CsPbIBr_2_ films.

It is well accepted that the PCEs of PSCs are determined by several crucial factors including the transport and recombination behavior of photogenerated carriers, defect density, sunlight absorption capability and energy level alignment between various films [67,68]. In order to elucidate the impacts of Cu^+^ substitution on the CsPbIBr_2_ film quality and the performance of CsPbIBr_2_-based PSCs, we employed XRD technique to investigate the crystallinity and the crystal structures of pristine CsPbIBr_2_ and various Cu^+^-doped CsPbIBr_2_ films. As depicted in Figure 2a, main characteristic peaks assigned to the α-phase CsPbIBr_2_ structure were observed in all samples, while no obvious impurity phases were found for all investigated films, revealing that Cu^+^ doping at different concentrations exhibited no obvious influences on the pure-phase cubic structure of CsPbIBr_2_. Based on the magnified XRD peaks in Figure 2b, the characteristic XRD peaks gradually shifted to higher angles with the increased Cu^+^ doping amounts, implying that the Cu^+^ cations with smaller ionic radius (0.60 Å) than that of Pb^2+^ (1.19 Å) were successfully doped in the lattice of CsPbIBr_2_ with a lattice contraction [69,70]. UV-vis absorption spectra were employed to evaluate the impacts of Cu^+^ substitution on the light absorption capability of CsPbIBr_2_ film. As depicted in Figure 2c, the Cu^+^ doping effectively increased the light absorption intensity of CsPbIBr_2_ film at 450–600 nm, especially for the CsPbIBr_2_-0.50%Cu film. Based on the Tauc plots of various films acquired from the UV-vis spectra in Figure S3, the band gap values of CsPbIBr_2_, CsPbIBr_2_-0.25%Cu, CsPbIBr_2_-0.50%Cu and CsPbIBr_2_-0.75%Cu were calculated to be 2.08, 2.07, 2.07 and 2.06 eV, respectively. It can be concluded that the Cu^+^ doping can improve the light absorption capability of CsPbIBr_2_ film, although the Cu^+^ doping displayed no obvious influences on the band gap values of CsPbIBr_2_, which may be beneficial for the PCE improvement of corresponding PSCs. Based on the steady-state PL spectra of various CsPbIBr_2_ films as depicted in Figure 2d, the Cu^+^-doped CsPbIBr_2_ films exhibited much lower PL intensity than the CsPbIBr_2_ film, demonstrating that the carrier recombination was effectively suppressed after introducing Cu^+^ cations to reduce the defect amount. Moreover, CsPbIBr_2_-0.75%Cu film with excessive Cu^+^ doping amount displayed a higher PL intensity than that of CsPbIBr_2_-0.50%Cu, implying more defects were formed in the CsPbIBr_2_-0.75%Cu film, agreeing well with the *J−V* results. It should be noted that the PL spectra of Cu^+^-doped CsPbIBr_2_ films exhibited several split peaks due to the phase segregation of CsPbIBr_2_ films, which may be beneficial for the PCE enhancement [62,71]. Based on the TRPL spectra as depicted in Figure S4, CsPbIBr_2_-0.50%Cu film delivered a much higher average carrier lifetime of 3.21 ns than the CsPbIBr_2_ film (0.88 ns) due to the effectively suppressed defect concentration, which was favorable for the transport and separation of carriers. XPS was further employed to explore the influences of Cu^+^ substitution on the chemical states of various ions in CsPbIBr_2_ films, with results displayed in Figure S5. All the XPS peaks of CsPbIBr_2_-0.50%Cu film shifted to lower binding energies compared with the CsPbIBr_2_ film due to the shrinkage of the BX_6_ octahedron caused by Cu^+^ doping induced by the changed interatomic force [58,72].

**Figure 3:**
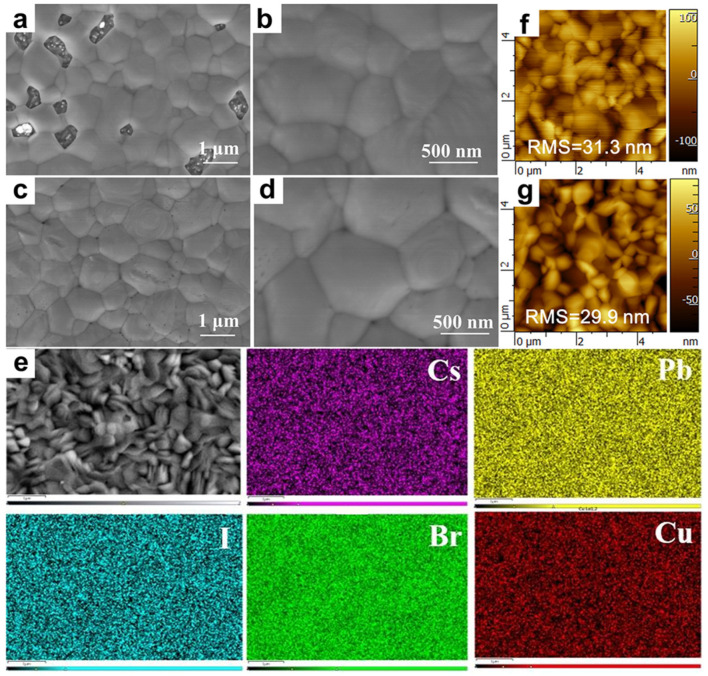
Top-view SEM images of (**a**,**b**) CsPbIBr_2_ and (**c**,**d**) CsPbIBr_2_-0.50%Cu films at different magnifications. (**e**) EDX elemental mapping images of CsPbIBr_2_-0.50%Cu film including Cs, Pb, I, Br, Cu elements. Top-view AFM images of (**f**) CsPbIBr_2_ and (**g**) CsPbIBr_2_-0.50%Cu films.

The morphology and quality of perovskite film plays a vital role in governing the performance of PSCs [54,73]. More specifically, compact perovskite films with large grain sizes and few grain boundaries are beneficial to suppress the carrier recombination [13,74]. Based on the top-view SEM images of CsPbIBr_2_ and various Cu^+^-doped CsPbIBr_2_ films in Figure 3a–d and Figure S6, the pristine CsPbIBr_2_ film displayed an inferior morphology with abundant pinholes and small grains, while the quality of CsPbIBr_2_ film was significantly improved after Cu^+^ doping in terms of reduced amount of pinholes and larger grain sizes. Particularly, the CsPbIBr_2_-0.50%Cu film showed a dense and uniform morphology with remarkably enlarged grain sizes and reduced amount of grain boundaries (Figure 3c,d). Nevertheless, CsPbIBr_2_-0.75%Cu film with excessive Cu^+^ doping amount exhibited smaller grain sizes than those of CsPbIBr_2_-0.50%Cu film, as displayed in Figure S6c,d. In addition, it was found that all elements were uniformly distributed on the CsPbIBr_2_-0.50%Cu film, as depicted in Figure 3e, demonstrating homogeneous distribution of Cu^+^ dopants. Based on the AFM images as depicted in Figure 3f,g and Figure S7, the CsPbIBr_2_-0.50%Cu film delivered a lower root-mean-square (RMS) surface roughness of 29.9 nm than the CsPbIBr_2_ film (31.3 nm), benefiting the interfacial charge transfer at perovskite film/HTL interface [75,76].

**Figure 4:**
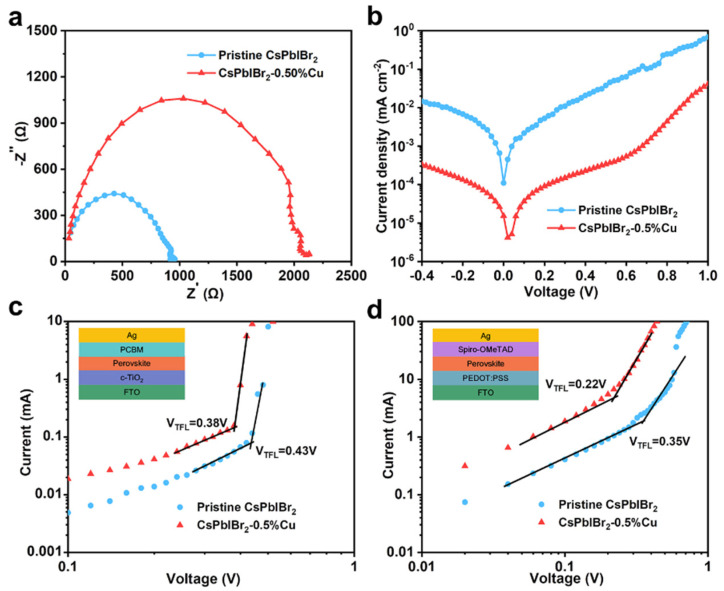
(**a**) EIS spectra and (**b**) *J−V* curves of CsPbIBr_2_ and CsPbIBr_2_-0.50%Cu cells in the dark. Dark *J−V* curves of (**c**) electron-only and (**d**) hole-only PSCs based on CsPbIBr_2_ and CsPbIBr_2_-0.50%Cu.

The carrier recombination behavior of CsPbIBr_2_ film after Cu^+^ doping was investigated by EIS at a fixed voltage of 0.5 V under dark conditions, as depicted in Figure 4a. As can be seen, a low-frequency arc corresponding to the recombination resistance (*R*_rec_) existed in the Nyquist plots, which was inversely proportional to the degree of carrier recombination at the interfaces between the perovskite films and TiO_2_/Spiro-OMeTAD layer [77,78]. The CsPbIBr_2_-0.50%Cu cell generated a larger *R*_rec_ value than the CsPbIBr_2_ cell, suggesting that the Cu^+^ doping remarkably suppressed the interfacial carrier recombination. Based on the dark *J−V* curves as displayed in Figure 4b, the CsPbIBr_2_-0.50%Cu cell delivered much smaller current densities than those of the pristine CsPbIBr_2_ cell, demonstrating that the photo-generated carriers were efficiently transported through charge-transporting layers of the CsPbIBr_2_-0.50%Cu cell instead of direct shunting, leading to reduced voltage/current loss and enhanced cell performance, which was attributed to the improved morphology and quality of CsPbIBr_2_-0.50%Cu films.

Hole-only and electron-only PSCs with structures of FTO/PEDOT:PSS/pervoskite/Spiro-OMeTAD/Ag and FTO/TiO_2_/pervoskite/[6,6]-phenyl-C61-butyric acid methyl ester (PCBM)/Ag were prepared to determine the trap densities of PSCs by measuring the dark *J−V* curves using the space charge-limiting current model [13,79]. As shown in Figure 4c,d, the traps were gradually filled until the applied voltage reached the trap-filling-limit voltage (*V*_TFL_). The trap densities (*N*_defects_) can be estimated by the equation of *N*_defects_ = $\frac{2\varepsilon \varepsilon_{0}V_{TFL}}{eL^{2}}$, where ε is the relative dielectric constant of CsPbIBr_2_ (approximately 8); ε_0_ is the vacuum dielectric constant; e represents elementary charge; and L is the thickness of CsPbIBr_2_ film (350 nm, Figure S1). The *V*_TFL_ values were 0.43 and 0.38 V for electron-only devices with CsPbIBr_2_ and CsPbIBr_2_-0.50%Cu films, corresponding to trap densities of 3.10 and 2.75 × 10^15^ cm^−3^, respectively. As for hole-only devices, the hole defect densities of CsPbIBr_2_ and CsPbIBr_2_-0.50%Cu films were 3.90 and 1.59 × 10^15^ cm^−3^ based on *V*_TFL_ values of 0.54 and 0.22 V, respectively. This suggested that the Cu^+^ doping effectively reduced the trapping centers for both electrons and holes of CsPbIBr_2_ film.

UPS technique was used to explore the effects of Cu^+^ substitution on the energy level alignment of CsPbIBr_2_-based PSCs. As displayed in Figure S8 and Table S2, the valence band maximum (*E*_VBM_) was calculated as −5.58 and −5.52 eV for CsPbIBr_2_ and CsPbIBr_2_-0.50%Cu, respectively, while the corresponding conduction band minimum (*E*_CBM_) was calculated to be −3.50 and −3.45 eV, respectively, based on the relationship of band gap = *E*_CBM_ − *E*_VBM_ [80]. In addition, the Fermi level of CsPbIBr_2_ was slightly increased from −3.74 to −3.68 eV after the introduction of 0.50% Cu^+^, which was closer to the CBM position (−3.51 eV) due to the n-type nature of Cu^+^-doped CsPbIBr_2_ [81]. The upshifted VBM position of CsPbIBr_2_-0.50%Cu effectively promoted the hole extraction from Spiro-OMeTAD to the perovskite layer. Moreover, the larger energy differences between the CBMs of CsPbIBr_2_-0.50%Cu and TiO_2_ may provide a higher driving force for the electron injection from light-absorbing film to the electron-transporting layer [72]. Furthermore, the hysteresis index of the CsPbIBr_2_ cell was reduced from 0.48 to 0.43 after the introduction of 0.50%Cu based on the *J−V* curves of corresponding PSCs under reverse and forward scan directions (Figure S9 and Table S3), which was attributed to the improved CsPbIBr_2_ film quality and promoted charge transfer, benefiting the cell stability.

**Figure 5:**
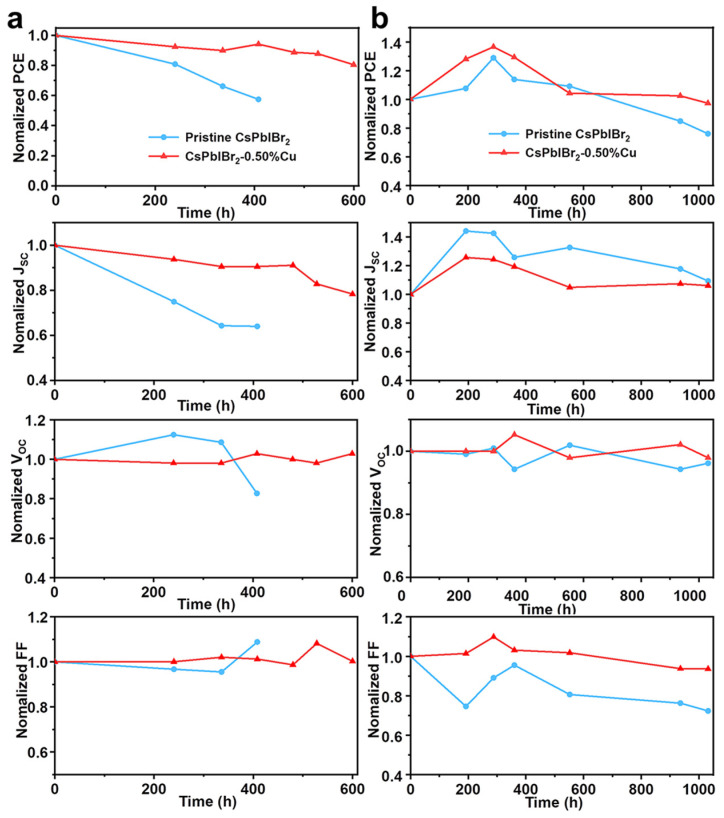
Normalized photovoltaic parameters of CsPbIBr_2_ and CsPbIBr_2_-0.50%Cu cells stored in humid air at (**a**) 25 °C and (**b**) 85 °C without encapsulation.

Besides the device efficiency, the humid and thermal stability is another crucial factor for the development of PSCs. As depicted in Figure 5a, the CsPbIBr_2_-0.50%Cu cell retained 94% of its primary PCE after storing in humid air with a relative humidity (RH) of 15–30% at 25 °C for 400 h, much superior to the pristine device (57%) under the same conditions. Furthermore, the PCE of the CsPbIBr_2_-0.50%Cu cell maintained 80% after 600 h storage in ambient condition with a RH of 15–30%. The high-temperature air stability of PSCs was evaluated by preparing HTL-free cells (FTO/c-TiO_2_/perovskite/carbon). The photovoltaic parameters of HTL-free CsPbIBr_2_ and CsPbIBr_2_-0.50%Cu cells are listed in Table S4. It was found that the CsPbIBr_2_-0.50%Cu cell delivered a superior PCE retention ratio of 97% to that of the CsPbIBr_2_ device (76%) after storing in ambient condition at 85 °C for 1000 h (Figure 5b).

Cu^+^ involved in the lattice of CsPbIBr_2_ partially substituted the Pb^2+^-occupied B-site, causing a lattice contraction, and resulted in a reduced bandgap for Cu^+^-doped CsPbIBr_2_ film. The reduced bandgap is beneficial for broadening light absorption band edge, thus facilitating the increase of photocurrent density. A previous study suggested that lattice contraction caused by doping could lead to improved heat stability for PSCs [82]. The presence of grain boundaries and pinholes might act as defect centers, trapping charge carriers, thereby reducing the PCE of PSC [83] Homogeneous and compact perovskite morphology with large grain sizes, less grain boundaries and pinholes enhanced the light capture of Cu^+^-doped perovskite film. The chemical bonds were enhanced after Cu^+^ doping, leading to higher phase stability [84]. Moreover, the defect density and the nonradiative recombination of the films were reduced.

## 4. Conclusions

In summary, we report a monovalent Cu^+^ cation doping approach to boost the PCEs and humid/thermal durability of CsPbIBr_2_-based all-inorganic PSCs. It was found that the introduction of a proper amount of Cu^+^ cations into CsPbIBr_2_ effectively passivated the defects, improved the perovskite film quality, suppressed the interfacial carrier recombination and enhanced the energy level alignment. Consequently, the optimized Cu^+^-doped cell exhibited a superb PCE of 9.11% with reduced hysteresis, which was 25.8% higher than that of pristine cell (7.24%). Moreover, the Cu^+^ doping also remarkably improved the thermal and humid stability of CsPbIBr_2_-based PSCs. For instance, the CsPbIBr_2_-0.50%Cu device retained 97% of the primary PCE after 1000 h storage at 85 °C in humid air, while the PCE of the pristine PSC declined to 76% under the same conditions. This work provides some important insights for the fabrication of durable CsPbIBr_2_-based all-inorganic PSCs with higher PCEs, which may promote the commercialization of this technology.

## Data Availability

The data that support the findings of this study are available from the corresponding author upon reasonable request.

## Supplementary Materials

The following supporting information can be downloaded at: https://www.mdpi.com/article/10.3390/nano12234317/s1, Figure S1: Cross-sectional SEM images of CsPbIBr_2_-based cell; Figure S2: (a) PCE, (b) *J*_sc_, (c) *V*_oc_ and (d) FF distributions of CsPbIBr_2_-based PSCs without and with 0.50% Cu^+^ doping with the corresponding average values shown inside; Figure S3: Tauc plots of CsPbIBr_2_ films with different Cu^+^ doping concentrations; Figure S4: TRPL spectra of CsPbIBr_2_ and CsPbIBr_2_-0.50%Cu films; Figure S5: XPS spectra of the pristine CsPbIBr_2_ and CsPbIBr_2_-0.50%Cu films: (a) Cs 3d, (b) Pb 4f, (c) I 3d, (d) Br 3d and (e) XPS survey spectra; Figure S6: Top-view SEM images of the (a, b) CsPbIBr_2_-0.25%Cu and (c, d) CsPbIBr_2_-0.75%Cu films; Figure S7: 3D-AFM models of (a) pristine CsPbIBr_2_ and (b) CsPbIBr_2_-0.50%Cu films; Figure S8: UPS spectra of pristine CsPbIBr_2_ and CsPbIBr_2_-0.50%Cu films: (a) the cut-off energies (Ecut-off) and (b) the positions of Fermi edge; Figure S9: *J*–*V* curves of (a) pristine CsPbIBr_2_ and (b) CsPbIBr_2_-0.50%Cu cells under revere and forward scan directions; Table S1: Photovoltaic performance parameters of CsPbIBr_2_-based PSCs with different Cu^+^ doping concentrations; Table S2: Calculated energy levels of pristine CsPbIBr_2_ and CsPbIBr_2_-0.50%Cu films; Table S3: Photovoltaic parameters of pristine CsPbIBr_2_ and CsPbIBr_2_-0.50%Cu cells under revere and forward scan directions; Table S4: Photovoltaic parameters of carbon-based HTL-free PSCs based on CsPbIBr_2_ and CsPbIBr_2_-0.50%Cu perovskites.
