# Supplementary material for: Monovalent Copper Cation Doping Enables High-Performance CsPbIBr2-Based All-Inorganic Perovskite Solar Cells
Source: Nanomaterials (Basel). 2022 Dec 5;12(23):4317. doi: 10.3390/nano12234317 (PMC9736419; doi:10.3390/nano12234317)
Supplement: Supplementary file 1 [file nanomaterials-12-04317-s001.zip › nanomaterials-2073565-supplementary.pdf]

Supporting Information

Monovalent Copper Cation Doping Enables High-Performance $CsPbIBr_2$-Based All-Inorganic Perovskite Solar Cells

Zhaonan Du [1,†], Huimin Xiang [1,†], Amin Xie [1], Ran Ran [1], Wei Zhou [1], Wei Wang [1,*] and Zongping Shao [2,*]

1 State Key Laboratory of Materials-Oriented Chemical Engineering, College of Chemical Engineering,
Nanjing Tech University, Nanjing 210009, China; 202161104046@njtech.edu.cn (Z.D.); 202062104009@njtech.edu.cn (H.X.); xam970922@163.com (A.X.); ranr@njtech.edu.cn (R.R.); zhouwei1982@njtech.edu.cn (W.Z.)

2 WA School of Mines: Minerals, Energy and Chemical Engineering, Curtin University, Perth, WA 6845, Australia

* Correspondence: wangwei@njtech.edu.cn (W.W.); zongping.shao@curtin.edu.au (Z.S.)

† These authors contributed equally to this work.

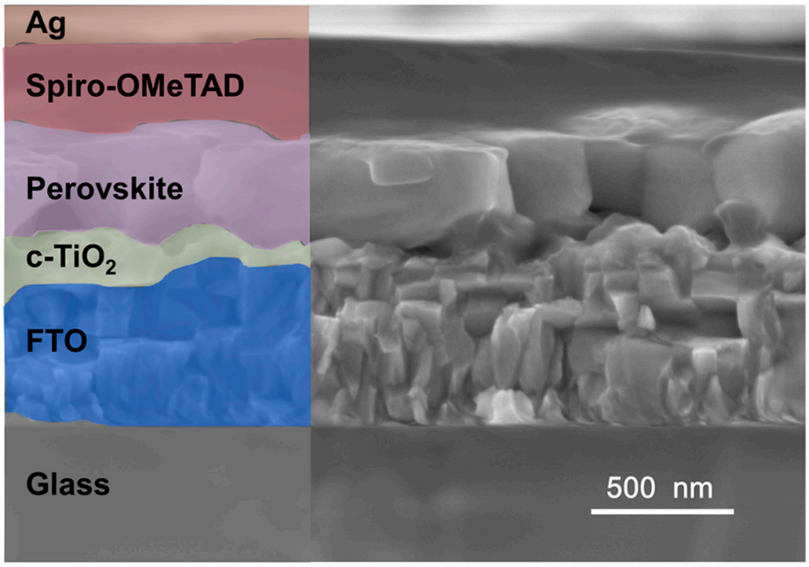

Figure S1. Cross-sectional SEM images of $CsPbIBr_2$-0.50%Cu cell.

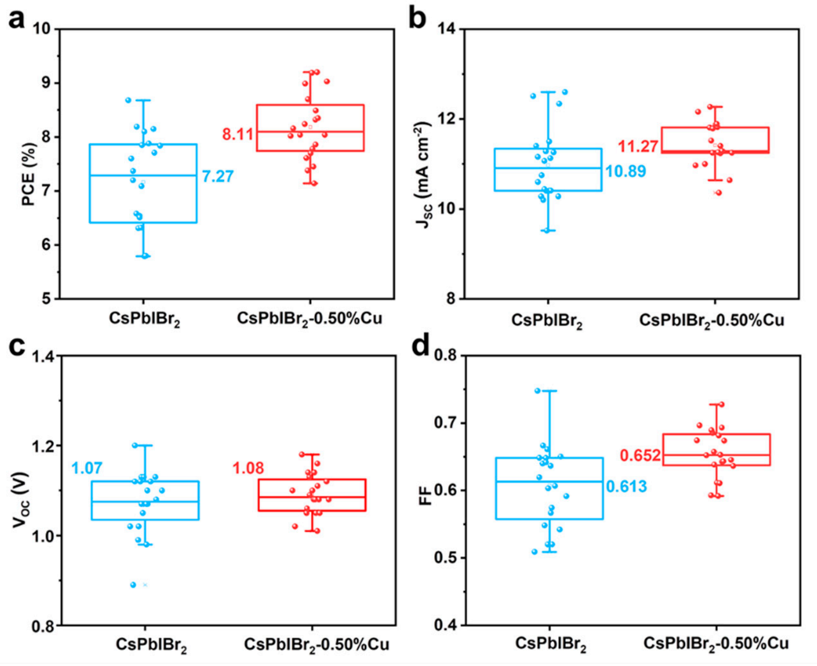

Figure S2. (a) PCE, (b) J_{sc}, (c) V_{oc} and (d) FF distributions of $CsPbIBr_2$-based PSCs without and with 0.50% Cu^+ doping with the corresponding average values shown inside.

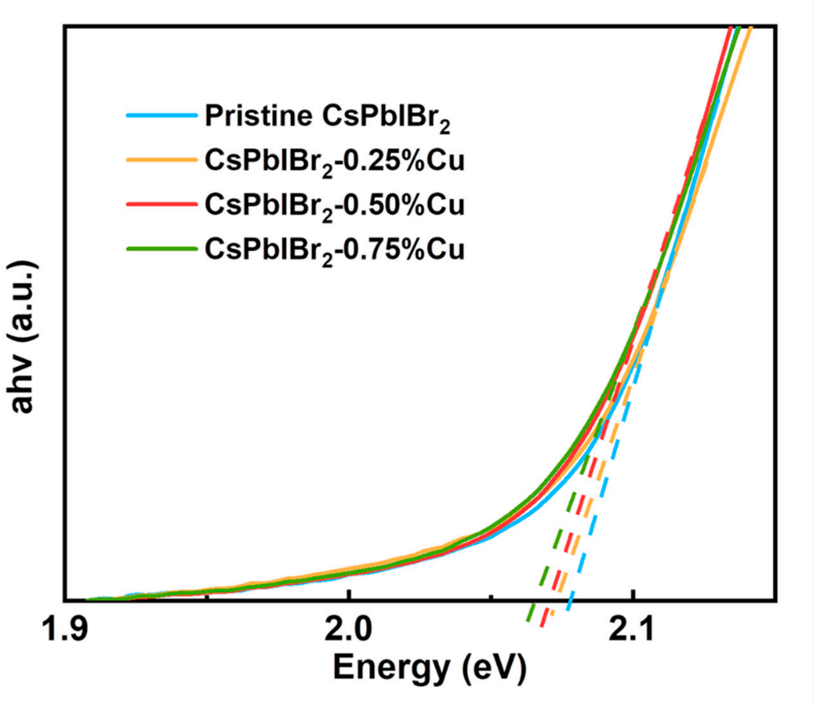

Figure S3. Tauc plots of $CsPbIBr_2$ films with different Cu^+ doping concentrations.

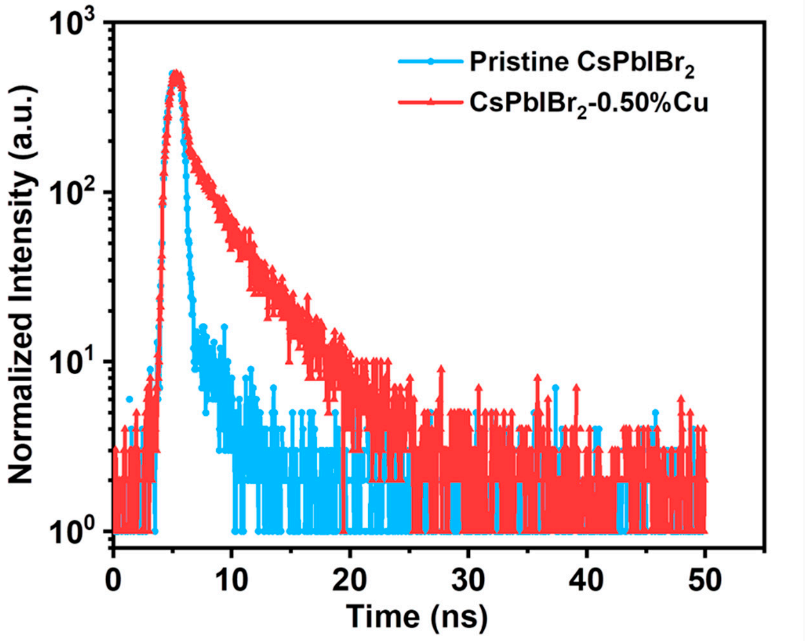

Figure S4. TRPL spectra of $CsPbIBr_2$ and $CsPbIBr_2$-0.50%Cu films.

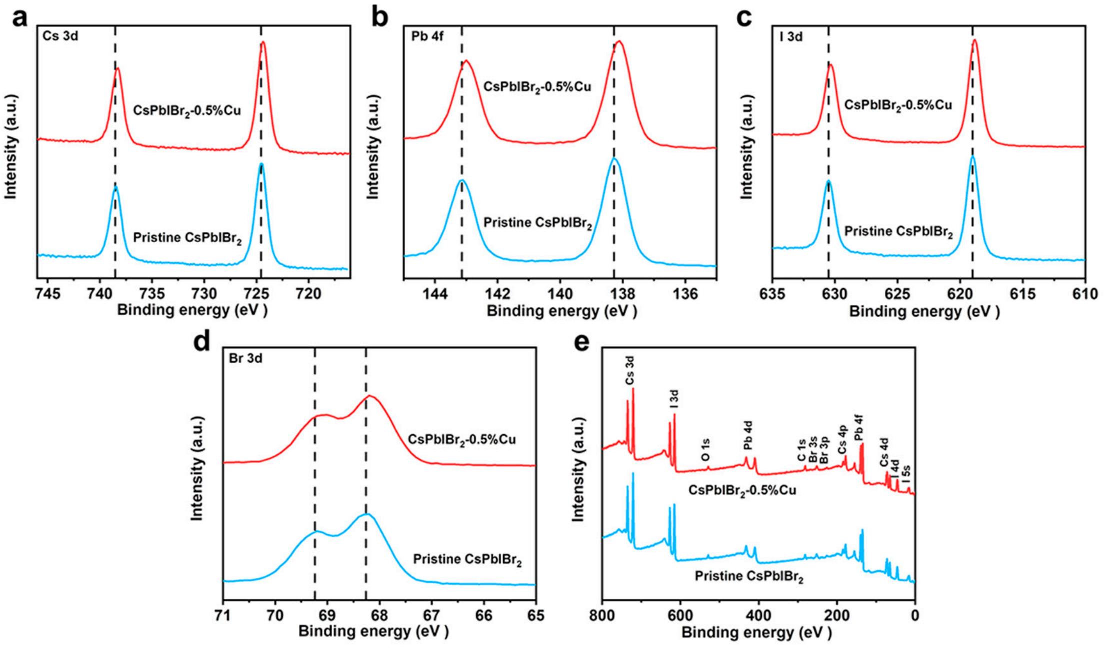

Figure S5. XPS spectra of the pristine $CsPbIBr_2$ and $CsPbIBr_2$-0.50%Cu films: (a) Cs 3d, (b) Pb 4f, (c) I 3d, (d) Br 3d and (e) XPS survey spectra.

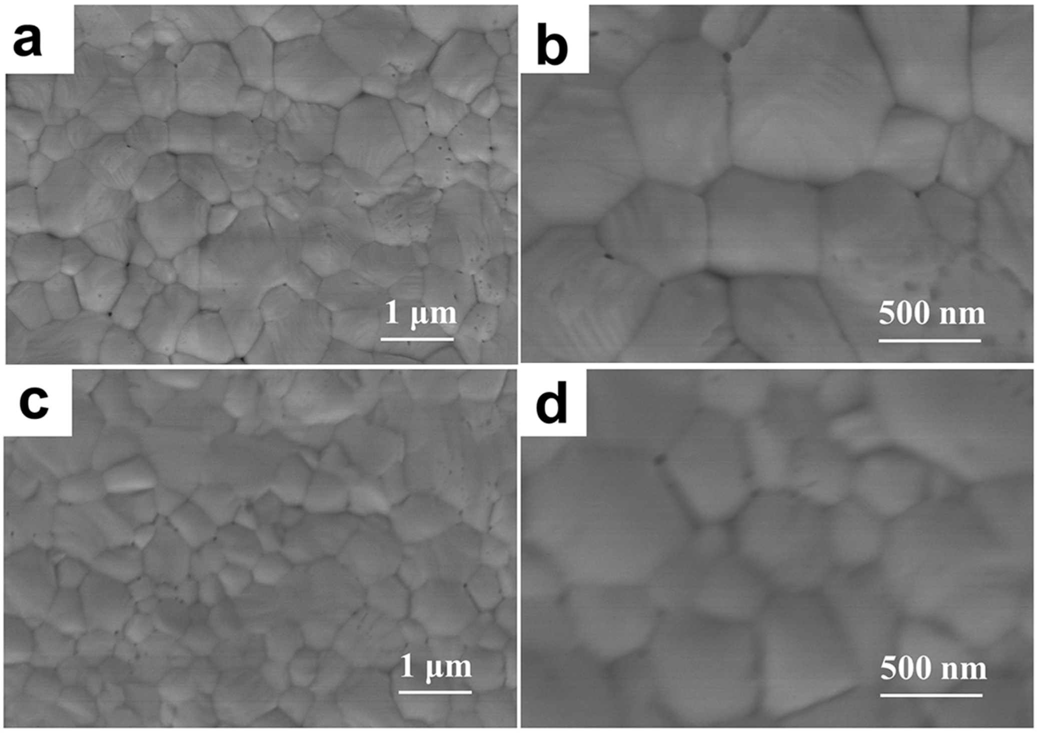

Figure S6. Top-view SEM images of the (a, b) $CsPbIBr_2$-0.25%Cu and (c, d) $CsPbIBr_2$-0.75%Cu films.

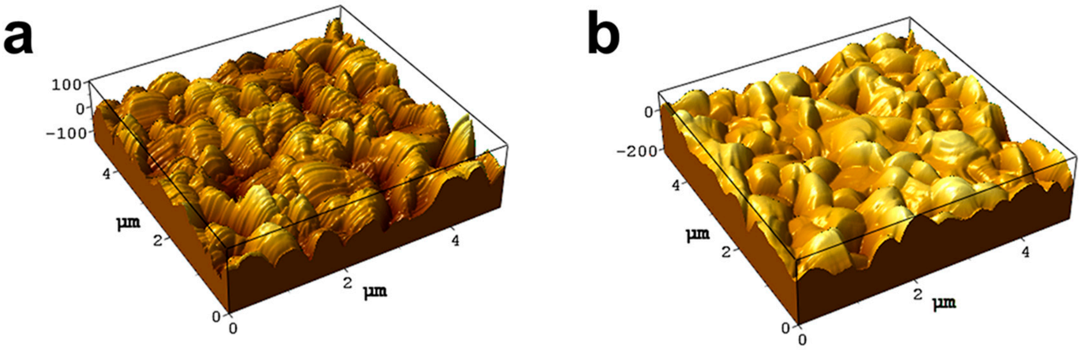

Figure S7. 3D-AFM models of (a) pristine $CsPbIBr_2$ and (b) $CsPbIBr_2$-0.50%Cu films.

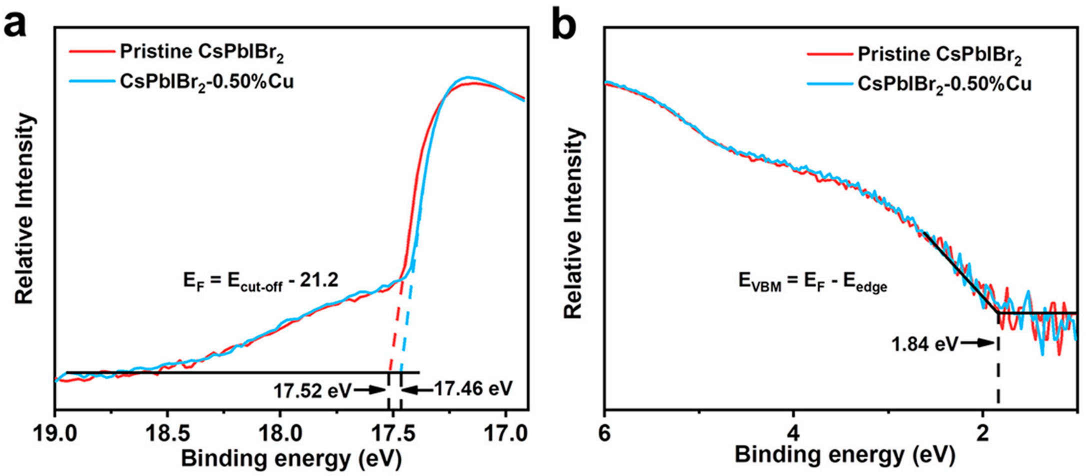

Figure S8. UPS spectra of pristine $CsPbIBr_2$ and $CsPbIBr_2$-0.50%Cu films: (a) the cut-off energies ($E_{cut\text{-}off}$) and (b) the positions of Fermi edge.

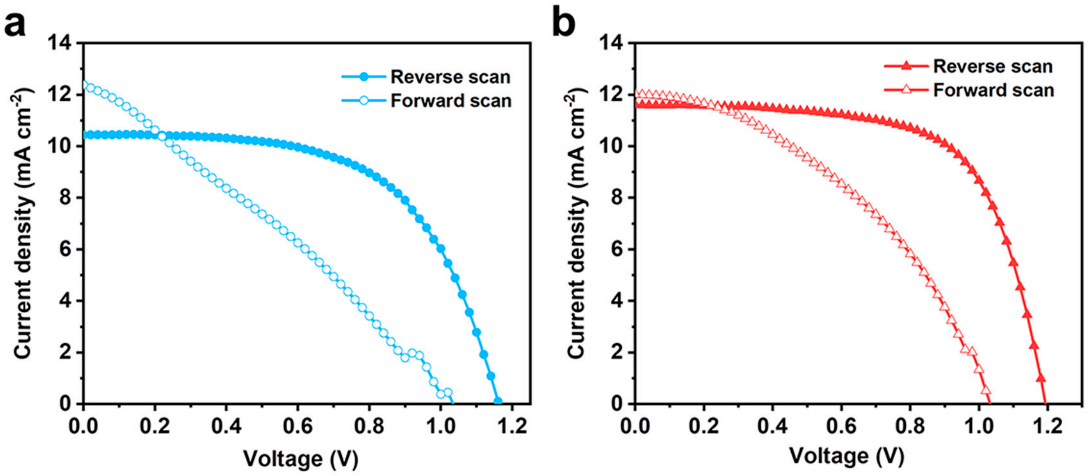

Figure S9. *J-V* curves of (a) pristine $CsPbIBr_2$ and (b) $CsPbIBr_2$-0.50%Cu cells under revere and forward scan directions.

Table S1. Photovoltaic performance parameters of $CsPbIBr_2$-based PSCs with different Cu^+ doping concentrations.

Sample	J_{sc} (mA cm^{-2})	V_{oc} (V)	FF	PCE (%)
Pristine $CsPbIBr_2$	10.4	1.16	0.597	7.24
$CsPbIBr_2$-0.25%Cu	11.1	1.18	0.620	8.34
$CsPbIBr_2$-0.50%Cu	11.6	1.19	0.658	9.11
$CsPbIBr_2$-0.75%Cu	12.1	1.19	0.620	8.91

Table S2. Calculated energy levels of pristine $CsPbIBr_2$ and $CsPbIBr_2$-0.50%Cu films.

Sample	Femi level (eV)	E_{VBM} (eV)	E_{CBM} (eV)	Band gap (eV)
Pristine $CsPbIBr_2$	-3.74	-5.58	-3.50	2.08
$CsPbIBr_2$-0.50%Cu	-3.68	-5.52	-3.45	2.07

Table S3. Photovoltaic parameters of pristine $CsPbIBr_2$ and $CsPbIBr_2$-0.50%Cu cells under revere and forward scan directions.

Sample	Scan direction	J_{sc} (mA cm^{-2})	V_{oc} (V)	FF	PCE (%)	HI
Pristine $CsPbIBr_2$	Reverse	10.4	1.16	0.597	7.24	0.48
	Forward	12.4	1.03	0.294	3.76	
$CsPbIBr_2$-0.50%Cu	Reverse	11.6	1.19	0.658	9.11	0.43
	Forward	12.0	1.03	0.418	5.18	

Table S4. Photovoltaic parameters of carbon-based HTL-free PSCs based on $CsPbIBr_2$ and $CsPbIBr_2$-0.50%Cu perovskites.

Sample	J_{sc} (mA cm^{-2})	V_{oc} (V)	FF	PCE (%)
Pristine $CsPbIBr_2$	11.25	0.99	0.425	4.74
$CsPbIBr_2$-0.50%Cu	11.64	1.11	0.483	6.23
